# The effectiveness of 6 versus 12-months of dialectical behaviour therapy for borderline personality disorder: the feasibility of a shorter treatment and evaluating responses (FASTER) trial protocol

**DOI:** 10.1186/s12888-018-1802-z

**Published:** 2018-07-17

**Authors:** Shelley F. McMain, Alexander L. Chapman, Janice R. Kuo, Tim Guimond, David L. Streiner, Katherine L. Dixon-Gordon, Wanrudee Isaranuwatchai, Jeffrey S. Hoch

**Affiliations:** 1 Centre for Addiction and Mental Health, 60 White Squirrel Way, Toronto, ON M6J 1H4 Canada; 2 Department of Psychiatry, University of Toronto, Toronto, ON Canada; 3 Department of Psychology, Simon Fraser University, 8888 University Drive, Burnaby, V5A 1S6 Canada; 4 DBT Centre of Vancouver, Vancouver, Canada; 5 Department of Psychology, Ryerson University, 350 Victoria Street, Toronto, ON M5B 2K3 Canada; 6 Department of Psychiatry and Behavioural Neurosciences, McMaster University, Box 585, 100 West 5th Street, Room B386, Hamilton, ON L8N 3K7 Canada; 7 Psychological and Brain Sciences, University of Massachusetts, 617 Tobin Hall, 135 Hicks Way, Amherst, MA 01003-9271 USA; 8 Centre for Excellence in Economic Analysis Research, St. Michael’s Hospital, 30 Bond Street, Toronto, ON M5B 1W8 Canada; 9 Institute of Health Policy, Management and Evaluation, University of Toronto, Toronto, Canada; 10 Department of Public Health Sciences, University of California, One Shields Avenue, Med Sci 1-C, Davis, CA 95616-8638 USA

**Keywords:** Borderline personality disorder, Self-injury, Suicide, Dialectical behaviour therapy, Randomized controlled trials

## Abstract

**Background:**

Although Dialectical Behaviour Therapy (DBT) is an evidence-based psychosocial treatment for borderline personality disorder (BPD), the demand for it exceeds available resources. The commonly researched 12-month version of DBT is lengthy; this can pose a barrier to its adoption in many health care settings. Further, there are no data on the optimal length of psychotherapy for BPD. The aim of this study is to examine the clinical and cost-effectiveness of 6 versus 12 months of DBT for chronically suicidal individuals with BPD. A second aim of this study is to determine which patients are as likely to benefit from shorter treatment as from longer treatment.

**Methods/Design:**

Powered for non-inferiority testing, this two-site single-blind trial involves the random assignment of 240 patients diagnosed with BPD to 6 or 12 months of standard DBT. The primary outcome is the frequency of suicidal or non-suicidal self-injurious episodes. Secondary outcomes include healthcare utilization, psychiatric and emotional symptoms, general and social functioning, and health status. Cost-effectiveness outcomes will include the cost of providing each treatment as well as health care and societal costs (e.g., missed work days and lost productivity). Assessments are scheduled at pretreatment and at 3-month intervals until 24 months.

**Discussion:**

This is the first study to directly examine the dose-effect of psychotherapy for chronically suicidal individuals diagnosed with BPD. Examining both clinical and cost effectiveness in 6 versus 12 months of DBT will produce answers to the question of how much treatment is good enough. Information from this study will help to guide decisions about the allocation of scarce treatment resources and recommendations about the benefits of briefer treatment.

**Trial registration:**

NCT02387736. Registered February 20, 2015.

## Background

Borderline personality disorder (BPD) is a serious and debilitating psychiatric condition affecting 1 to 6% of the population [1, 2]. Of particular concern, BPD is associated with exceedingly high rates (80%) of self-injury [3] and suicide-related mortality (9–33%) [4, 5] comprising 28–47% of all mortalities by suicide [6–8]. Notably, individuals with BPD are overrepresented in primary care [9] and mental health care settings [10, 11]. As such, BPD is a serious health concern that heavily taxes the mental health system [12–14]. Furthermore, individuals with BPD frequently have difficulties with social and occupational functioning [15], and disproportionately utilize social assistance [16]. Together, these factors make BPD a particularly costly disorder to treat [17–19].

Among the psychosocial treatments showing efficacy for BPD [20–23], dialectical behaviour therapy (DBT), a comprehensive cognitive behavioural treatment, has accrued the most empirical support [24]. DBT involves weekly hour-long *individual therapy*, weekly *group skills training* (typically 2–2.5 h), between-session *telephone consultation* as needed to coach the patient in the use of behavioural skills (typically by phone or other communication media), and weekly *therapist consultation team meetings* designed to support, motivate, and enhance the skills of therapists [25]. Several randomized controlled trials have evaluated a standard 12-month version of DBT. Results support the effectiveness of DBT relative to treatment as usual for reducing self-injurious behaviours and treatment dropout among BPD patients [26–28]. As well, one trial demonstrated the superiority of DBT to non-behavioural treatment by experts in terms of reducing self-injurious behaviours, treatment dropouts, and hospitalizations among suicidal patients with BPD [29]. In addition, DBT has shown comparable efficacy to other structured BPD-specific treatments [21, 30]. Recent meta-analyses demonstrate that DBT is associated with medium to large effects in terms of improvements in self-injurious behaviours [31, 32], anger, and overall mental health [31]. Standard DBT is lengthy and resource intensive [33] but it is associated with reduced overall cost burden associated with BPD [34–36]. Due to its robust support, international guidelines for effective psychosocial treatment have identified DBT as a treatment for BPD that has accumulated the most evidence, particularly for individuals with self-injurious behaviours [37, 38].

Although DBT is effective [31, 32], and cost-effective [34, 36], the treatment remains lengthy (often 12 months or longer) and comprehensive, requiring substantial resources to implement. Public health care systems often lack the resources to develop and sustain DBT programs [39, 40]; thus, the vast majority of individuals with BPD are left without access to evidence-based treatments. Furthermore, private insurance often does not fully cover comprehensive DBT [41]. Accordingly, many treatment centres implement idiosyncratic, truncated versions of standard DBT [42–44] that do not have the empirical support of standard 1-year DBT [45]. The potential cost-effectiveness and ease of delivery for brief DBT (defined here as 6-months) would allow effective treatment to reach larger numbers of patients. Consequently, clinicians and researchers have identified the need for brief, effective treatments for BPD as an urgent health priority [43].

Despite the dearth of research on the optimal duration of specialized treatments for BPD such as DBT, there is some evidence indicating that brief forms of DBT are effective. Only one randomized controlled trial (RCT) to date has examined 6-months of standard DBT [33], focusing on female veterans with BPD. Compared with 6-months of treatment as usual, patients who received 6-months of DBT exhibited greater improvements in suicidal ideation, hopelessness, depression, and anger expression [33]. This study, however, was relatively small (*n* = 10 per arm) and did not compare 6-months of DBT with a longer course of DBT. More recently, findings from a larger (*n* = 45 per arm) non-randomized trial suggested that 6-months of DBT for adults with BPD yielded improvements in self-harm, emergency department visits, hospitalizations, depression, anxiety, and overall functioning, relative to a treatment as usual wait list condition [36]. Similarly, a small (*n* = 20) uncontrolled study examined a 6-month version of standard DBT for suicidal patients with BPD, and found excellent treatment retention (95%) and significant pre- to post-treatment reductions in self-harm, suicidal ideation, depression, and hopelessness [46]. Thus, emerging data suggest that 6-month is a promising duration for a briefer course of DBT.

Despite the urgent need to identify whether briefer versions of DBT are clinically and cost effective for individuals diagnosed with BPD, research in this area is limited. First, extant studies of brief DBT have been small and underpowered. Second, there is a dearth of RCTs, making it hard to draw conclusions about the findings. Third, no studies have directly compared briefer forms of DBT with the most commonly used duration in the literature (12 months). Finally, the health economic impact of 6-months of DBT has not been evaluated. Although the direct costs of implementing 6-months of DBT will inevitably be lower compared with 12-months, the long-term costs or savings associated with 6-months of treatment require investigation.

Further, given the considerable heterogeneity among the symptoms, characteristics, and presenting problems of BPD patients, variability in treatment response is inevitable. Mean effect sizes for each group will likely over- or underestimate the response to treatment for some patients [47], and the identification of predictors of treatment response is critical to informing the effectiveness and efficiency of therapies [48]. Therefore, it is important to examine which patients do achieve superior benefits from 6-months versus 12-months of DBT.

Few studies have examined predictors of treatment outcomes for BPD patients. Although the treatments in these studies did not include standard DBT and involved acute (5-day [43]) or brief (≤5-month) adjunctive treatments [49–51], several patient characteristics were associated with outcome. For instance, high levels of clinical severity predicted greater improvement in BPD symptoms [49] and high levels of impulsivity and self-harm frequency [43, 50] predicted greater improvements in self-harm although it’s unclear whether these findings may be due to a regression to the mean. Additionally, co-occurring generalized anxiety disorder (GAD) predicted less improvement in self-harm and other self-destructive behaviours [50], and cluster A personality disorders predicted less improvement in self-destructive behaviours [50] and depression [51]. Furthermore, co-occurring post-traumatic stress disorder (PTSD) and cluster C personality disorders predicted less improvement of emotion dysregulation and quality of life [50]. Taken together with the promising findings for 6-month versions of DBT in the treatment of BPD patients with high rates self-harm and impulsive behaviours, these data suggest that some BPD patients may not achieve sufficient benefits from brief treatments. Predictors of poorer response to brief DBT may include the presence of co-occurring PTSD, GAD, and cluster A or C personality disorders. Clarifying how different lengths of DBT work for subsets of patients with BPD will have a major impact on how healthcare resources may be allocated and patients triaged.

In sum, though DBT is the most empirically-supported psychosocial treatment for BPD, the 12-month version of DBT studied most often in clinical trials requires substantial resources. Currently, the optimal “dose” of treatment is unknown. The present study will fill an important gap in this research, by (1) comparing the benefits of 6-months versus 12-months of DBT in a rigorous RCT for BPD patients with chronic self-injury, (2) examining the economic impact of 6- versus 12-months of DBT, and (3) identifying patient characteristics that differentiate which individuals do and do not benefit from 6-months of DBT. With a focus on high risk individuals with BPD, such research will answer the question of whether and for whom 6-months of DBT is clinically indicated and/or economically attractive.

## Methods/design

### Study aims and hypotheses

#### Aim 1

To evaluate the clinical effectiveness of DBT-6 compared to DBT-12 for the treatment of chronically self-harming individuals with BPD. The following main hypotheses will be examined:

**Hypothesis 1a**: Patients in the DBT-6 arm will show equivalent reductions in the frequency and severity of self-harm across the treatment phase compared with patients in the DBT-12 arm. **Hypothesis 1b**: Patients in the DBT-6 arm will show equivalent reductions in the frequency and severity of self-harm across a 1-year post treatment follow-up phase, compared with patients in the DBT-12 arm.

#### Aim 2

To identify which subtypes of BPD patients are as likely to benefit from DBT-6 versus DBT-12.

**Hypothesis 2a:** Patients who present with high rates of self-harm and impulsive behaviours will have reductions in the frequency and severity of self-harm behaviours that are comparable in the DBT-6 arm and the DBT-12 arm, over the course of both the treatment phase and the 1-year post treatment follow-up.

**Hypothesis 2b**: Patients with co-occurring post-traumatic stress disorder, generalized anxiety disorder, or cluster A or C personality disorders will exhibit lesser reductions in the frequency and severity of self-harm behaviours in the DBT-6 arm compared to the DBT-12 arm, over the course of both the treatment phase and the 1-year post treatment follow-up. In other words, a subtype by duration interaction is expected.

### Study design

This is a two-site, single-blind, two-arm randomized controlled trial. To date, study enrollment is complete. Two-hundred and forty participants were randomly assigned to receive either 12-months of DBT (DBT-12) or 6-months of DBT (DBT-6). Follow-up assessments are occurring every 3 months, with the final assessment occurring at 24-months.

### Study setting and recruitment

The study is being conducted at two sites, the BPD Clinic at the Centre for Addiction and Mental Health (CAMH) in Toronto, Ontario, and the Personality and Emotion Research Laboratory in conjunction with the DBT Centre in Vancouver, British Columbia. Of the 240 participants, 160 were enrolled in Toronto and another 80 participants were enrolled at the Vancouver site. Participants were drawn from existing treatment or research wait-lists at the respective sites, through advertisements at hospitals, universities, and health service centres, and via word of mouth referrals from clinicians seeing potentially appropriate patients.

Prospective participants were pre-screened on the phone by a research assistant. The research assistant described the study, explained the screening process, and informed potential participants that if found eligible, they would be randomized to either 12 or 6-months of DBT. If the individual was interested, the research assistant conducted a telephone screen to gather information pertinent to inclusion and exclusion criteria. Individuals who met eligibility criteria were invited to attend an in-person assessment session to determine their eligibility. The in-person assessment was conducted by trained study assessors, who were responsible for obtaining prospective participant consent to participate in the trial. These assessors were also responsible for administering structured interviews and tests that focused on inclusion/exclusion criteria. If the participant was deemed eligible for participation, the interview continued to include structured diagnostic interviewing of Diagnostic and Statistical Manual-IV Axis I and II disorders. The in-person assessment, conducted over the course of 2 days included laboratory measures of attention and implicit associations of emotion regulation with self-harm and the completion of self-report questionnaires. All procedures were approved by CAMH and SFU research ethics boards.

### Participants

#### Inclusion criteria

Individuals aged 18–65 were eligible to participate if they (a) met DSM-IV criteria for BPD based on the International Personality Disorders Examination (IPDE), (b) exhibited recent and chronic self-injurious behaviours, operationalized as at least 2 episodes of self-injury or suicide attempts in the past 5 years, including at least 1 episode in the past 8 weeks, (c) are proficient in English, (d) consent to study participation, (e) have not received more than 8 weeks of standard DBT in the past year, and (f) have either Ontario Health Insurance Plan (OHIP) coverage or BC Medical Services Plan (MSP) health insurance for 1 year or more.

#### Exclusion criteria

Participants were excluded from the study if they (a) met the criteria for a specific psychotic disorder, bipolar disorder I, or dementia, based on the DSM-IV [52], (b) have an estimated IQ less than or equal to 70, (c) have a chronic or serious physical health problem expected to require hospitalization within the next year (e.g., cancer), or (d) have plans to move out of the province in the next 2 years.

#### Assessment of inclusion/exclusion criteria

Assessors masked to condition assignment and calibrated with a gold-standard assessor on study instruments are assessing participants’ symptoms of DSM-IV diagnoses. BPD criteria are being assessed with the International Personality Disorder Examination [53], a well-established semi-structured interview used by the World Health Organization. Other personality disorders are being assessed with the Structured Clinical Interview for the DSM-IV, Axis II (SCID-II [54]). DSM-IV Axis I disorders will be assessed with the SCID-I (for Axis I disorders), Patient Version ([55]). Cognitive functioning is being assessed with the Wechsler Test of Adult Reading (WTAR) [56], a brief measure of verbal intelligence.

#### Randomization

Individuals who provided informed consent and were eligible were informed of the treatment arm to which they were assigned by each site’s coordinator after the baseline assessment. The allocation scheme was developed in order to maintain blindness and random assignment, while minimizing wait-list time and avoiding unfilled slots in either treatment arm. The allocation scheme used variable block sizes with permutations of 4 and was generated by the statistician. A research assistant then put the allocation scheme into a series of consecutively numbered white, opaque, sealed envelopes (numbered 1 to n, where n is the study site sample size). When there was a minimum of 1 opening per each of the 2 treatment conditions at either treatment site, the research coordinator opened the next randomization envelope, which revealed participant assignment to either 6-months (DBT-6) or 12-months (DBT-12) of DBT. Randomization was conducted independently at each site.

### Interventions

We are comparing two interventions: DBT 12-months [25, 57, 58] and DBT 6-months.

**DBT** is a comprehensive therapy that blends acceptance-based techniques derived from the Zen tradition [25, 57–59] with strategies from traditional cognitive behavioural therapy (CBT), including problem-solving, behavioural analysis, contingency management, and skills training techniques. DBT consisted of: 1) weekly 1-h individual therapy session, 2) a 2-hour weekly skills training group, 3) access to 24 h/7 days a week telephone consultation and, 4) weekly therapist consultation meetings.

#### Comparison of DBT-6 and DBT-12

The treatment conditions are comparable on all factors except for length of treatment. Both treatment conditions involve all four treatment components, and an equal number of treatment hours per week. In order to control for hours of therapy received, participants are expected to not engage in other primary psychosocial treatments. Further, to control for possible confounding effects of therapist characteristics, therapists across the 2 conditions are matched on a number of factors including expertise, training in DBT, and availability of supervision.

#### Treatment dropouts

Consistent with the DBT treatment protocol, participants who fail to attend four consecutive scheduled individual or group sessions are discontinued from treatment and will be considered dropouts.

#### Therapists

Therapists at both sites include doctoral and master’s level therapists who have attended formal DBT basic and advanced-level workshops, with a minimum of 2 years of supervised experience in DBT and treating BPD patients. Senior therapists (SM, JK, AC) who are certified DBT practitioners with the Linehan Board of Certification and Accreditation are supervising the therapists and leading supervision and therapist consultation meetings at each site.

### Treatment adherence

Therapist competence and treatment delivery is being monitored via therapist adherence ratings, individual supervision (weekly for students and unregistered clinicians who require more monitoring), and weekly DBT consultation team meetings. All individual and group therapy sessions are being videotaped, and therapist adherence is being assessed using the University of Washington DBT Adherence Rating Scale [60]. This scale provides scores on a scale of 0 to 5 across a range of DBT strategies [25, 57–59], with global adherence scores of ≥4.0 indicating adherence to DBT. Psychology graduate student coders masked to treatment assignment and trained to an acceptable level of reliability at the University of Washington in Seattle, WA are independently rating a random selection of 5% of sessions from each therapist-patient dyad, with an equal proportion of sessions coded in the pre-treatment orientation (first 4 weeks), early, middle, and late stages of treatment. As well, 5% of all group sessions are being evaluated for adherence.

### Assessments

Over the course of the study (i.e., 6 or 12 month treatment and follow-up phase), participants are being assessed at 9 time points: pretreatment, 3, 6, 9, 12, 15, 18, 21 and 24 months. Outcome measures are the same as those used in previous RCTs of DBT [27–30, 61, 62], allowing for comparability with previous outcomes. The measures are described below, and Table 1 summarizes the schedule of measures.

**Table 1:** Summary of measures

Domain	Report type	Outcome Variable	Measure	Screen	Baseline	3 months	6 months	9 months	12 months	15 months	18 months	21 months	24 months	Post session 1–4	Monthly
Suicide/Self-Harm	Self-Report	Suicidal Ideation	Beck Scale for Suicide Ideation (BSS)		•	•	•	•	•	•	•	•	•		
	Interview	Lifetime Suicide and Self-Injury	Lifetime Suicide Attempt Self-Injury Interview (LSASI)		•										
		Suicide and Self-Injury (past 3 months)	Suicide Attempt Self-Injury Interview (SASII)		•	•	•	•	•	•	•	•	•		
Symptoms	Self-Report	Depression	Beck Depression Inventory-II (BDI-II)		•	•	•	•	•	•	•	•	•		
		Impulsivity	Barratt Impulsiveness Scale-11 (BIS-11)		•	•	•	•	•	•	•	•	•		
		Borderline Personality Disorder (BPD)	Borderline Symptom List-23 (BSL-23)		•	•	•	•	•	•	•	•	•		
		Emotion Regulation Difficulties	The Difficulties in Emotion Regulation Scale (DERS)		•	•	•	•	•	•	•	•	•		
		Interpersonal Problems	Inventory of Interpersonal Problems-64 (IIP-64)		•	•	•	•	•	•	•	•	•		
		Non-Suicidal Self-Injury	Inventory of Statements About Self-Injury (ISAS)		•										
		Post-Traumatic Stress Disorder	PTSD Checklist for DSM-5 (PCL-5)		•	•	•	•	•	•	•	•	•		
		Psychiatric Symptom Distress	Symptoms Checklist-90-Revised (SCL-90-R)		•	•	•	•	•	•	•	•	•		
		Anger Experience and Expression	State and Trait Anger Expression Inventory-2 (STAXI-2)		•	•	•	•	•	•	•	•	•		
		Alcohol Use and Related Problems	The Alcohol Use Disorder Identification Test (AUDIT)		•	•	•	•	•	•	•	•	•		
		Drug Use and Related Problems	Drug Abuse Screening Test (DAST)		•	•	•	•	•	•	•	•	•		
		Childhood Trauma	Childhood Trauma Questionnaire Short Form (CTQ-SF)			•									
		Five Dimensions of Personality	The NEO Five Factor Personality Inventory Short Form (NEO-SF)		•										
Functioning	Self-Report	Social Functioning	Social Adjustment Scale Self-Report (SAS-SR)		•	•	•	•	•	•	•	•	•		
		Medical Outcome	Medical Outcome Score-Short Form (MOS-SF-36)		•	•	•	•	•	•	•	•	•		
		Quality of Life	Euroqol-5D-5 Level (EQ-5D-5 L)		•	•	•	•	•	•	•	•	•		
Healthcare	Interview	Healthcare Utilization	Treatment History Interview (THI-2)		•	•	•	•	•	•	•	•	•		
Descriptive	Interview	Demographics	Demographic Data Survey (DDS)	•											
		Premorbid IQ	Weschler Test of Adult Reading (WTAR)	•											
Skills	Self-Report	Dialectical Behavior Therapy Skills Use	The DBT Ways of Coping Checklist (DBT-WCCL)		•	•	•	•	•	•	•	•	•		
		Mindfulness Skills	Kentucky Inventory of Mindfulness Skills (KIMS)		•	•	•	•	•	•	•	•	•		
Mental Disorders	Interview	BPD Diagnosis	Int’l Personality Disorder Examination - BPD Section (IPDE-BPD)	•									•		
		Psychiatric Disorders	Structured Clinical Interview for DSM-IV-TR Axis I Disorders (SCID I)	•^a^	•										
		Personality Disorders	Structured Clinical Interview for DSM-IV-TR Axis II Disorders (SCID II)		•										
Treatment Appraisal	Self-Report	Expectancy and Credibility of Treatment	The Credibility/Expectancy Questionnaire (CEQ)		•										
		Reasons for Early Treatment Termination	Research for Early Termination from Treatment - Client (RET-C)^b^												
Alliance	Self-Report	Therapeutic Alliance	Working Alliance Inventory - Short Form, Client (WAI-S)			•	•							•	
		Session Ruptures and Resolutions	Post Session Questionnaire - Client (PSQ)											•	
Therapist Measures	Self-Report	Therapeutic Alliance	Working Alliance Inventory - Short Form, Therapist (WAI-S)			•	•							•	
		Session Ruptures and Resolutions	Post Session Questionnaire - Therapist (PSQ)											•	
		Initial Expectation of Treatment Helpfulness	Expectations for Treatment - Therapist Version 4 Item (ET-T)											•^c^	
		Expectation of Treatment Helpfulness	Expectations for Treatment - Therapist Version 5 Item (ET-T)			•	•^d^								
		Therapist Demographics	Therapist Demographic Questionnaire^e^												
		Mindfulness Skills	Kentucky Inventory of Mindfulness Skills–Therapist (KIMS)												•
		Job-Related Stress and Burnout	Maslach’s Burnout Inventory-Therapist Version (MBI)												•

^a^Only Psychotic Symptoms and Mania Sections of the SCID are completed at In-Person Screen

^b^Completed by participant in case of early termination of treatment

^c^Initial Expectations for Treatment Version 4 Item completed only after Session 1

^d^Expectations for Treatment Version 5 Item completed at 6-months only for participants receiving 12 months of treatment

^e^Completed by therapist prior to their first participant

### Clinical outcomes

#### Primary outcome

The primary outcome is the frequency and severity of self-injurious episodes, measured by the Suicide Attempt Self-Injury Interview (SASII [63]). The SASII is a semi-structured interview that measures frequency and medical severity of self-injury and suicide attempts and is administered by trained assessors at all assessment points to measure self-injury over the previous 3-month assessment interval.

#### Secondary outcomes/predictors of response

Secondary outcomes and predictors of response measures assess a range of characteristics and suicidal and self-harm behaviours, health-related outcomes, including hospitalization, emergency room visits, psychiatric symptoms, social functioning, general functioning, and treatment retention. Characteristics of self-injury (including suicide attempts and non-suicidal self-injury), including frequency, medical severity, intent to die, lethality, and precipitants of self-injury, are being assessed with the Lifetime Suicide Attempt Self-Injury Count (L-SASI) (Lifetime Parasuicide History; Linehan & Comtois, unpublished 1996). See Table 1 for schedule of measures. Healthcare utilization is being measured with the Treatment History Interview − 2 (THI-2) (THI; Linehan & Heard, unpublished 1987), which assesses the number and type of outpatient psychosocial treatments, number and duration of hospital admissions, frequency of emergency department visits, and medication use. Borderline personality disorder severity is being assessed using the Borderline Symptom List – 23 (BSL-23 [64, 65]), a self-report measure, assessing the severity of BPD symptoms in the past week. Impulsivity is being assessed using the Barratt Impulsiveness Scale (BIS-11 [66]), and depressive symptoms are being measured using the Beck Depression Inventory-II (BDI-II [67]). The State-Trait Anger Expression Inventory-2 (STAXI-2 [68]) is being used to measure participants’ experiences and expressions of anger. The Symptom Checklist-90 Revised (SCL-90-R [69]), a widely used self-report questionnaire, is being used to measure past-week general symptom distress. Interpersonal functioning is being assessed with the Inventory of Interpersonal Problems-64 (IIP-64 [70]), which assesses dysfunctional patterns in interpersonal interactions. Social functioning is being assessed with the Social Adjustment Scale-Self Report (SAS-SR [71]), which measures social adjustment. Health-related quality of life outcomes are being monitored using the EuroQol 5D-5 L (EQ-5D-5 L [72]) and the Medical Outcomes Score Short Form (SF-36 [73]), which assesses physical and mental health functioning. The Alcohol Use Disorders Identification Test (AUDIT [74]) is a 10-item screening questionnaire regarding the amount and frequency of alcohol consumption, dependence on alcohol, and problems associated with alcohol use, and is being used to assess for alcohol-related problems. The Drug Abuse Screening Test (DAST [75]), a 28-item self-report questionnaire widely used in treatment evaluation research, is being used to assess problems associated with drug misuse. The Inventory of Statements About Self-Injury (ISAS [76]), is a self-report questionnaire that measures non-suicidal self-harm behaviors. Suicidal intentions are assessed using the Beck Scale for Suicidal Ideation (BSS [77]), a self-report questionnaire consisting of 19 items. The PTSD Checklist for DSM-5 (PCL-5 [78]) is a 20-item self-report measure used to assess symptoms of posttraumatic stress disorder. Emotion dysregulation is measured using the Difficulties in Emotion Regulation Scale (DERS [79]), a 36-item, self-report. Mindfulness is being assessed with the Kentucky Inventory of Mindfulness Skills (KIMS [80]) self-report questionnaire. The Dialectical Behavior Therapy-Ways of Coping Checklist (DBT-WCCL [81]) is used to assess thoughts and behaviors related to coping strategies during stressful events.

The following other measures are administered to assess predictors of response. Demographic information will be collected with the Demographic Data Schedule (DDS [82]) at baseline. The NEO-Five Factor Inventory, short form (NEO-SF [83]), a widely used personality measure, is being used to assess personality dimensions (i.e., neuroticism, extraversion, openness, agreeableness, and conscientiousness) and is collected at baseline. The Childhood Trauma Questionnaire-Short Form (CTQ-SF [84]), a self-report measure of childhood abuse and neglect is being collected at 3-months and the Credibility/Expectancy Questionnaire (CEQ [85]) is being collected at baseline. The Working Alliance Inventory-Short Form (WAI-S [86]), therapist and client version are collected after each of the first 4 treatment sessions and at 3 and 6-months.

Finally, the Reasons for Early Termination from Treatment Questionnaire (RET-C [87]) is administered to evaluate reasons for premature termination and is administered to participants who drop out of treatment.

Participants are being compensated at a rate of $10 Canadian dollars (CDN) per hour for the completion of these study measures.

#### Health economic outcomes

One objective of this research is to compare the cost of DBT-6 vs DBT-12. Costs include direct treatment cost, health services cost (e.g., hospitalization, emergency room visits, day surgery or procedure, physician visits, medications), productivity costs, and law enforcement and related cost. The cost analysis will be based on the healthcare resources data obtained from the THI-2 in the trial and with participant consent, imported to and linked with federal and provincial administrative databases (for Ontario, the Institute for Clinical Evaluative Sciences; for British Columbia, B.C. Population Health; in addition to the Canadian Institute for Health Information). Additionally, using established guidelines for cost-effectiveness research [88], data on therapist and consultation team time devoted to research participants (e.g., in meetings, individual and group sessions, between-session communication) are being collected and used to calculate per patient cost of service provision based on expected service provider costs (e.g., salary, benefits).

#### Genetic data

An amendment to the original protocol for the addition of a genetic component to the study and collection of saliva samples was approved in September, 2015. A separate consent form was developed for this data collection. Participants’ who provide consent to the genetic study are required to provide five small saliva samples (about half a teaspoon each) to the research team at the following time points: a) at baseline, b) at 6-months, c) 1 year, d) one-and-a-half years, and e) 2 years into the study. These time-based samples will be used specifically for epigenetic comparisons before, during and after DBT treatment. Patients are receiving $20 remuneration per saliva sample.

Data collection for the study began in February, 2015 and is expected to be complete by July, 2019.

Interview outcome data will be double data-entered. Other data collected via an electronic encrypted data management system are not double data entered. Data is stored on an encrypted server. Study outcome data is stored in a locked cabinet in accordance with ethical guidelines governing the management of data. All study data collected will be maintained for 25 years before being destroyed.

### Masking of treatment allocation

Several methods will be used to conceal treatment allocation of participants and protect against sources of bias. Therapists will not function as assessors, and vice versa. Study assessors are masked to participants’ treatment assignment with the exception of assessors who administer the Treatment History Interview-2 (THI-2) (THI; Linehan & Heard, unpublished 1987). This measure includes questions about the treatment received and may disclose the condition to which the participant is assigned, therefore an RA who is not masked to the participant’s treatment assignment will conduct this interview. Third, both therapists and assessors will consistently be reminded of the study masking requirements, and discussions about clients between therapists and assessors will be discouraged.

### Sample size and power analysis

The study was designed to test whether DBT-6 is not inferior to DBT-12 (an established treatment). The estimate of the sample size required for a non-inferiority test was based on the effect sizes from prior DBT RCTs [30, 62]. Post-treatment suicide and self-harm estimates over 4 months were 2.26 episodes for DBT-6 and 0.73 for DBT-12 (SD_pooled_ = 4). We expected no significant differences between the DBT-6 and DBT-12 conditions in terms of reductions in the frequency and severity of self-injury episodes at 1 year. We therefore defined our delta, the clinically significant range of indifference for our non-inferiority trial, as a difference in outcomes of 1.53 (SD = .04) episodes in the frequency of self-harm episodes at post-treatment. To achieve this maximum of 1.53 or less in the difference of self-injury rates with adequate power (α = .05; 1 - β = .80), 85 participants per group are required to show non-inferiority of treatment. Allowing for a 30% dropout rate, we will recruit 120 participants per group. The sample size calculation also addresses the necessary power for secondary analyses, given that the sample size required for null hypothesis significance testing is typically less than those required for non-inferiority [89].

### Statistical analyses

#### Baseline characteristics

We will examine rates of ineligibility. Demographic and clinical characteristics will be summarized using descriptive statistics.

#### Primary analyses

##### Comparison of DBT-6 versus DBT-12

Analyses will be conducted at the end of treatment and at the end of follow-up. No interim analyses are planned. The outcome analyses will be conducted on an intention-to-treat basis. The primary outcomes, frequency and severity of suicide and self-harm behaviours, are both expected to have a skewed distribution. The count nature of the self-injury frequency variable, as well as the self-injury severity ratings, are integers bounded by zero. As well, given the dependencies of the observations over time and individual variability, our primary analyses will employ a multilevel random effects Poisson growth curve model. The occasion level random effects will capture secondary over-dispersion due to the heterogeneity of observations within participant self-injury reporting [88, 90]. These random effects will follow a log-normal distribution [91], and will reduce or eliminate the disturbance effects associated with the differences between rates and forms of specific, within individual, self-harm that can bias estimates of true self-harm rates.

Specific tests of our primary hypotheses (non-inferiority tests of equivalence and post hoc comparisons of difference) will be model-based [92] using contrasting means and regression coefficients. Evaluation of model fit will be provided by statistical indices including Akaike’s Information Criteria (AIC) and Bayesian Information Criteria (BIC) and likelihood ratio tests. To perform the comparison of self-injury outcomes at post-treatment (our primary research interest), 1-sided non-inferiority t-tests of the marginal means will be performed. If missing data are at moderate levels or are related to a specific set of covariates or outcome, we will compare multiple imputation [93], growth curve analysis [94], and instrumental variables analysis [95] since no one technique is demonstrably superior [96]. We will also examine differences in sites and therapist characteristics that may be evidenced in treatment outcomes.

#### Health economic analyses

We will compare the cost of DBT-6 vs DBT-12 from the perspective of the public healthcare payer. The output will be the incremental cost of DBT-6 compared to DBT-12. We will analyze the total cost as a dependent variable, using a regression model to estimate the difference in expected healthcare cost between the two groups. The intervention will be the primary independent variable and the regression model will adjust for potential confounding variables. In theory, an ordinary least squares model produces unbiased estimates even if the data are skewed; however, additional estimation methods (e.g., generalized linear models) and different uncertainty methods (e.g., parametric and non-parametric bootstrapping) will be explored to facilitate investigation of the impact of various cost assumptions [97–99].

Additionally, as a secondary economic analysis, we will explore the economic evaluation using the net benefit regression framework [100]. The outcome will be the incremental net benefit from DBT-6 for the intervention group compared to the standard DBT-12 group. We will also estimate the incremental cost per one self-injury episode avoided and the incremental cost per quality-adjusted life-year (QALY) gained, derived from the SF-36 and EQ-5D-5 L data. To estimate QALYs gained, we will convert SF-36 and EQ-5D-5 L data collected in the trial to utility scores using a validated algorithm [101–103]. The QALY is the gold standard measure of effectiveness recommended for economic evaluation and allows for a more global measure of the impact of a clinical intervention. Calculating QALYs requires the combination of health-related quality of life measures with data on health state duration. The EQ-5D-5 L has been used to evaluate the quality of life of patients with schizophrenia [104] and depression [105]; however, recently its validity in BPD studies has been questioned, so the SF-36 will be used for comparison in a sensitivity analysis. Statistical uncertainty will be characterized using a 95% confidence interval and a cost-effectiveness acceptability curve (CEAC) [106].

#### Attrition and treatment implementation

We will examine rates of treatment completion and attrition across both groups. We will conduct survival analyses to examine differences in the timing of treatment dropouts. In addition, we will compare rates of use of psychotropic medications and other adjunctive treatments at baseline and across both treatments. Treatment adherence ratings across both treatment arms will be evaluated. We also will examine potential site differences between the CAMH and SFU sites in terms of participant characteristics, and attrition. We will also examine potential therapist effects on treatment implementation and dropout rates.

#### Secondary analysis

##### Analyses of subtypes of BPD patients likely to benefit from DBT-6 versus DBT-12

Analyses related to predictors of treatment response will involve growth curve modeling, with covariates, with the two arms using both linear and over-dispersed Poisson hierarchical models. The effects of impulsivity and the rates of self-injury will be managed by person centering the variables. Growth curve models will be used to estimate the trajectories of the 4 diagnostic groups that are expected to moderate treatment response (PTSD, GAD, Cluster A, and Cluster C), but without the inclusion of covariates, provided they are not needed. Each diagnostic category will be estimated independently within a single model. The tests of both slopes and marginal effects will be carried out post-estimation, and will involve pair-wise multiple comparisons with alpha levels that are Holm’s Sequential Bonferroni adjusted.

### Data safety monitoring

A data safety monitoring committee (DSMC) was established and composed of three independent researchers specializing in BPD and self-harm, cognitive behavior therapy, and biostatistics. The DSMC is responsible for monitoring the research protocol, reviewing data, assessing the safety of the trial, reporting adverse events, reviewing unanticipated problems, and monitoring protocol violations in accordance with the policies and procedures outlined in the institutional research ethics boards (REBs) at each site and in accordance with Canadian Institutes of Health Research’s (CIHR) policies and procedures concerning data and safety monitoring. The DSMC will communicate any new information to the REB at both sites over the course of the trial, and the site supervisors (SM, AC), in consultation with the research team will make the decision whether to continue, suspend, modify or stop the trial, or amend the protocol. The site supervisors (SM, AC) are responsible for monitoring and reporting serious adverse events to the DSMC. Serious adverse events are defined as any untoward medical occurrence that results in death, is life-threatening, results in persistent or significant disability/incapacity, results in a congenital anomaly/birth defect, or any important medical event that may jeopardize the health of the research participant or may require medical intervention to prevent one of the outcomes listed above. Serious adverse events involving self-harm behaviour was operationalized as very high to severe medical risk according to the SASII lethality scale.

## Discussion

This will be the first rigorously controlled trial comparing two different lengths of DBT for individuals with BPD and chronic suicidal behaviour. Given the severity [4, 5] and societal costs of BPD [17–19], and the limited resources available for psychotherapy for BPD [39, 40], findings have the potential to significantly impact clinical practice. If 6- months of DBT produces comparable clinical outcomes to 1-year of DBT, the briefer version would be an excellent, less resource intensive alternative that could help to increase access to treatment, reducing wait times and enable more people to be treated. If 6 months of DBT became the new standard length of treatment, it would reduce the direct costs and resources compared with 1-year. If the study hypotheses are confirmed, the findings could encourage decision-makers to invest in the development of briefer programs, improving treatment accessibility.

Limitations of the study include the following: This study design does not include a control arm that could control for the passage of time. Though this would provide a more rigorous test of our study hypotheses, a third control arm (e.g., wait-list or treatment as usual) was ruled out due to ethical concerns and because of the strong evidence base demonstrating that standard DBT-12 is superior to unstructured treatment as usual controls. As well, concomitant psychotropic medications will be uncontrolled. While psychotropic utilization is a potential confound in the current study design, the restriction of medications would reduce referrals to the study and also pose a threat to external validity. Indeed, previous trials indicate that an estimated 80% of patients will be on at least one psychotropic medication [107, 108] and thus, restricting medication use would compromise the representativeness of the study sample. Therefore, by monitoring medication use throughout the trial, we believe that the current study balances the internal and external validity in a manner that is the best way to advance treatment of this population.

### Study timeframe

Study enrollment began in February, 2015 and the total sample of 240 participants was completed by June, 2017. The active treatment phase is expected to be finished in June, 2018. The completion of follow-up assessments is expected in July, 2019. The dissemination of final results at international meetings is expected by the Fall of 2019. Publication of the findings is planned for 2020.

## Abbreviations

AIC: Akaike’s information criteria
AUDIT: Alcohol use disorders identification test
BDI-II: Beck Depression Inventory-II
BIC: Bayesian information criteria
BIS-II: Barratt impulsiveness scale
BPD: Borderline personality disorder
BSL-23: Borderline Symptom List-23
BSS: Beck scale for suicidal ideation
CAMH: Centre for addiction and mental health
CBT: Cognitive behavioural therapy
CDN: Canadian
CEQ: Credibility/Expectancy Questionnaire
CIHR: Canadian Institutes of Health Research
Cluster A: Paranoid, Schizoid, Schizotypal
Cluster C: Avoidant, Dependent; Obsessive compulsive
CTQ-SF: Childhood Trauma Questionnaire-Short Form
DAST: Drug abuse screening test
DBT: Dialectical behavior therapy
DBT-WCCL: Dialectical Behavior Therapy-Ways of Coping Checklist
DDS: Demographic data schedule
DERS: Difficulties in emotion regulation scale
DSMC: Data safety monitoring committee
DSM-IV: Diagnostic and Statistical Manual of Mental Disorders, 4th edition
EQ-5D-5L: EuroQol 5D-5L
FASTER: Feasibility of a shorter treatment and evaluating responses
GAD: Generalized anxiety disorder
IIP-64: Inventory of Interpersonal Problems-64
IPDE: International personality disorders examination
IQ: Intelligence quotient
ISAS: Inventory of Statements About Self-Injury
KIMS: Kentucky inventory of mindfulness skills
L-SASI: Lifetime parasuicide history
MSP: Medical services plan
NEO-SF: NEO-Five Factor Inventory, short form
OHIP: Ontario Health Insurance Plan
PCL-5: PTSD Checklist for DSM-5
PTSD: Post-traumatic stress disorder
QALY: Quality-adjusted life-year
RCT: Randomized controlled trial
RET-C: Reasons for early termination from treatment questionnaire
SASII: Suicide attempt self-injury interview
SAS-SR: Social adjustment scale-self report
SCID-I: Structured Clinical Interview for DSM-IV Axis I Personality Disorders
SCID-II: Structured Clinical Interview for DSM-IV Axis II Personality Disorders
SCL-90-R: Symptom Checklist-90 Revised
SF-36: Medical Outcomes score short form
SFU: Simon Fraser University
STAXI-2: State-Trait Anger Expression Inventory-2
THI-2: Treatment History Interview − 2
WA: Washington
WAI-S: Working Alliance Inventory-Short Form
WTAR: Wechsler Test of Adult Reading
